# Serum Sodium Concentration and Increased Risk for Primary Epiretinal Membrane

**DOI:** 10.3389/fmed.2021.770362

**Published:** 2021-12-24

**Authors:** Can Can Xue, Jing Cui, Xiao Bo Zhu, Jie Xu, Chun Zhang, Dong Ning Chen, Ya Xing Wang, Jost B. Jonas

**Affiliations:** ^1^Department of Ophthalmology, Peking University Third Hospital, Beijing, China; ^2^Beijing Ophthalmology and Visual Sciences Key Laboratory, Beijing Institute of Ophthalmology, Beijing Tongren Hospital, Capital Medical University, Beijing, China; ^3^Department of Physical Examination, Beijing Tongren Hospital, Capital Medical University, Beijing, China; ^4^Department of Ophthalmology, Dongfang Hospital, Beijing University of Chinese Medicine, Beijing, China; ^5^Medical Faculty Mannheim, Department of Ophthalmology, Heidelberg University, Mannheim, Germany; ^6^Institute of Clinical and Scientific Ophthalmology and Acupuncture Jonas & Panda, Heidelberg, Germany; ^7^Institute of Molecular and Clinical Ophthalmology, Basel, Switzerland

**Keywords:** epiretinal membrane, associated factors, serum sodium, prevalence, epidemiology

## Abstract

**Aims:** To examine the prevalence of primary epiretinal membranes (ERMs) and associated systemic factors.

**Methods:** The cross-sectional, community-based Tongren Health Care Study enrolled participants who received regular health examinations in the Beijing Tongren Hospital from 2017 to 2019. Using fundus photographs, retinal specialists assessed the presence of ERMs and their systemic associations.

**Results:** Primary ERMs were detected in 841/22820 individuals, with a prevalence of 3.7% [95% confidence intervals (CI): 3.4–3.9%] in the total study population (mean age: 44.5 ± 13.8 years) and 6.5% (95% CI: 6.1–7.0%) in individuals aged 40+ years. In multivariable analysis, a higher ERMs prevalence was associated with older age [odds ratio (OR): 1.10; *P* < 0.001], higher serum cholesterol concentration (OR: 1.14; *P* = 0.003) and higher serum sodium concentration (SSC) (OR: 1.12; *P* < 0.001). In women, a higher SSC, even within the normal range, was associated with an increased risk of ERMs (OR: 1.19; *P* < 0.001). Female participants with an SSC of 144–145mmol/L as compared with those with an SSC of 135–137 mmol/L had a 5-fold increased odds of having ERMs (All women: OR: 5.33; *P* < 0.001; Women aged 40+years: OR: 4.63; *P* < 0.001).

**Conclusion:** Besides older age and higher serum cholesterol concentration, a higher SSC, even if within the normal range, was independently associated with a higher ERM prevalence in women.

## Introduction

Epiretinal membranes (ERMs) are a common cause of visual impairment in the elderly population (1–5), with the pooled prevalence from population-based studies to be 9.7% (6). While intraocular causes including retinal diseases, previous intraocular surgeries, hyperopia, or myopia were reported to be associated with secondary ERMs, to mention a few (1, 3, 5, 7, 8), idiopathic ERMs present in eyes without evident abnormality, and their causes remain to be elusive (9). Systemic factors associated with the presence of ERMs were older age, female sex, ethnic background, hyperlipidemia, smoking, diabetes mellitus, and a lower serum concentration of urine acid (1, 3, 4, 7, 10, 11), however, none of the above-mentioned factors were consistently reported besides the increasing age.

Most ERMs remain relatively stable and treatment is not required, while vitrectomy surgery is indicated for ERMs patients associated with symptoms that severely affect their activities of daily living, including decreased visual acuity, metamorphopsia, double vision, or difficulty using their eyes together (2, 12). Of particular note is that even a very successful vitrectomy does not always ensure a desirable visual outcome (2). Our knowledge on the pathophysiology of ERM has been improved greatly by histopathological studies and advanced image technologies, however, there're no preventive measures since the exact pathological mechanisms of ERMs remain unknown.

The current community-based large-scale study was conducted in 20,000+ participants with comprehensive general medical examinations, with the aim to explore systemic factors associated with the primary ERMs.

## Methods

The cross-sectional, community-based Tongren Health Care Study included individuals who attended regular health care check-up examinations in the Beijing Tongren Hospital from July 2017 to December 2019. The study population has been described in detail recently (13). The eligibility criterion for inclusion into the current study was an age of 18+ years. The study was conducted in adherence to the Declaration of Helsinki and approved by the Medical Ethics Committee of Beijing Tongren Hospital.

The examinations included an interview in which general demographic data and information about the medical history and other health-related events were obtained. The physical examinations included the assessment of anthropometric parameters such as body height and weight and circumferences of the waist and hip, measurement of blood pressure and electrocardiography, and biochemical examinations of samples of blood. Arterial hypertension was defined as a systolic blood pressure of 140 mmHg or more, a diastolic blood pressure of 90 mmHg or more, or a physician's-based diagnosis of hypertension or use of antihypertensive medication. Diabetes mellitus was defined based on the history of a previously diagnosed diabetes or receiving a glucose-lowering therapy and/or fasting blood glucose concentration of ≥7.0 mmol/L. The estimated glomerular filtration rate (eGFR) was calculated based on the serum creatinine concentration and using the Chronic Kidney Disease Epidemiology Collaboration equation (14).

The ophthalmological examinations, performed by experienced ophthalmologists, consisted of the measurement of best-corrected visual acuity, non-contact tonometry, slit lamp-based biomorphometry of the anterior and posterior segment of the eye, and 45° non-mydriatic fundus photography (Topcon TRG-NW7SF, Topcon, Tokyo, Japan; or Cannon, CR6-45NM, Canon, Tokyo, Japan). Based on the assessment of the fundus photographs, an ERM was classified into either a less severe subtype termed as cellophane macular reflex (CMR) or a more severe form termed as preretinal macular fibrosis (PMF) (3, 15). Eyes with the coexistence of CMR and PMF were classified as having PMF. A secondary ERM was defined as being associated with intraocular diseases including diabetic retinopathy, late-stage age-related macular degeneration, other retinopathy, and previous intraocular surgery including cataract surgery or vitreous retinal surgery (3). All ERMs without any associated intraocular disorder were classified as primary ERMs.

The data were analyzed using the statistical software SPSS 26.0 (SPSS Inc., Chicago, IL, USA) and the Statistical Package for the R (version 1.4.1103). Continuous variables were presented as mean ± standard deviation and categorical variables as the number of cases/percentages. In the univariable analysis, a binary logistic regression analysis was performed to assess associations between the ERM prevalence and other systemic parameters, without and with adjustment for age, the odds ratios (ORs) and their 95% confidence intervals (CI) were calculated. The multivariable analysis was then performed with the ERM prevalence as the dependent variable, and all those parameters with *P*-values ≤ 0.10 in the age-adjusted analysis as the independent variables, in all participants, in men and in women, respectively. The collinearity was considered when a variance inflation factor (VIF) of more than 5 was observed. After detecting the association between serum sodium concentration (SSC) and ERM, we re-assessed our findings by excluding individuals with hypernatremia (SSC > 145 mmol/L) and hyponatremia (SSC <135 mmol/L) to eliminate their confounding effects. We compared the SSCs among different age groups in the whole participants, in men and women respectively, using one-way analysis of variance (ANOVA) and adjusted by Bonferroni when a significant difference was detected. We stratified the SSC into four groups (135.0–137.0, 138.0–140.0, 141.0–143.0, and 144.0–145.0 mmol/L) and assessed its association with the ERM prevalence. A three-dimensional surface plot was created to visualize the associations among age, SSC, and the presence of ERM for women aged 40+ years, as most epidemiologic studies included individuals aged 40+ years old. A two-tailed *P*-value of < 0.05 was considered statistically significant.

## Results

Out of 22,945 individuals (12,574 men, 54.8%) who were examined during the study period, 125 (0.54%) participants were excluded due to unreadable fundus photographs. The study eventually included 22,820 participants (12,514 women, 54.8%) with a mean age of 44.5 ± 13.8 years (range: 18–97 years). The mean uncorrected visual acuity, best-corrected visual acuity, and intraocular pressure were 0.40 ± 0.40 logarithm of the minimal angle of resolution (logMAR), 0.04 ± 0.10 logMAR, and 14.3 ± 3.0 mmHg, respectively.

### Prevalence of Epiretinal Membranes

ERMs were found in 988 participants (4.3 ± 0.1%; 95% CI: 4.1–4.6%), among whom 147 subjects (0.6 ± 0.1%; 95% CI: 0.5–0.7%) with diabetic retinopathy (19/12.9%), retinal vascular diseases (7/4.8%), late-stage age-related macular degeneration (6/4.1%), other types of retinopathies (20/13.6%), history or signs of vitreous or retinal surgery (7/4.7%), and previous cataract surgery (88/59.9%) were classified as secondary ERMs and were excluded.

Primary ERMs (described as “ERMs” in the following paragraphs) were detected in 841 [345 (41.0%) men] out of the 22,820 individuals, with a prevalence of 3.7 ± 0.1% (95% CI: 3.4–3.9%). Unilateral ERMs and bilateral ERMs were found in 618 participants (73.5%) and 223 subjects (26.5%), respectively. For individuals aged 40+ years, the prevalence of ERMs was 6.5 ± 0.2% (95% CI: 6.1–7.0%), 5.8 ± 0.3% (95% CI: 5.2–6.4%), and 7.2 ± 0.3% (95% CI: 6.6–7.9%) for all individuals, for men, and for women, respectively (Table 1).

**Table 1:** Prevalence of primary epiretinal membranes (ERMs) in all, in men and in women.

**Age group (years)**	**All**		**Men**		**Women**	
	***n***	**%**	***n***	**%**	***n***	**%**
<40	17/10,196	0.2 (0.1, 0.3)	7/4,453	0.2 (0.05, 0.3)	10/5,743	0.2 (0.1, 0.3)
40–49	40/4,805	0.8 (0.6, 1.1)	17/2,242	0.8 (0.4, 1.1)	23/2,563	0.9 (0.5, 1.3)
50–59	221/4,118	5.4 (4.7, 6.1)	85/1,933	4.4 (3.6, 5.4)	136/2,185	6.2 (5.2, 7.5)
60–69	436/2,736	15.9 (14.7, 17.7)	170/1,239	13.7 (11.9, 16.3)	266/1,497	17.8 (16.0, 20.5)
70+	127/965	13.2 (11.3, 16.0)	66/439	15.0 (11.8, 21.5)	61/526	11.6 (9.1, 15.5)
Total	841/22,820	3.7 (3.4, 3.9)	345/10,306	3.3 (3.0, 3.7)	496/12,514	4.0 (3.6, 4.3)

*The prevalence of primary ERMs is shown as the number of patients / all study participants and mean (95% confidence interval)*.

### Serum Sodium Concentration as a Risk Factor for Epiretinal Membranes

In the univariable analysis, a higher ERM prevalence was associated with older age, female sex, higher prevalence of hypertension and diabetes, higher body mass index, waist-hip circumference ratio, systolic and diastolic blood pressure, SSC, higher serum concentrations of potassium, blood urea nitrogen, glucose, high sensitive C-reactive protein, lipoprotein a, triglycerides, total cholesterol, and low-density lipoprotein, and lower serum concentrations of calcium, phosphate and lower eGFR (all *P* ≤ 0.05) (Table 2). In the multivariable analysis, a higher ERM prevalence remained to be significantly associated with older age (OR: 1.10; *P* < 0.001), higher SSC (OR: 1.12; *P* < 0.001), higher total cholesterol (OR: 1.14; *P* = 0.003), and borderline with eGFR (OR: 1.01; *P* = 0.05) for all participants. A higher ERM prevalence was consistently associated with increasing age, in men and women, respectively, whereas its association with higher SSC and higher total cholesterol only remained statistically significant in women but not men (Table 3).

**Table 2:** Factors associated with primary epiretinal membranes (ERMs) by univariable logistic analysis and age-adjusted logistic analysis.

	**No-ERMs**	**Primary ERMs**	**Univariable analysis**		**Age-adjusted analysis**	
	***N* = 21,832**	***N* = 841**	***P-*value**	**OR (95%CI)**	***P-*value**	**OR (95%CI)**
Age (year)	43.6 ± 13.3	62.4 ± 8.7	<0.001	1.10 (1.09, 1.11)		
Women (*n*/%)	11,940/54.7	496/59.0	0.014	1.19 (1.04, 1.37)	0.002	1.26 (1.09, 1.46)
Body mass index (kg/m^2^)	23.9 ± 3.7	24.3 ± 3.1	0.001	1.03 (1.01, 1.05)	0.552	0.99 (0.97, 1.02)
Waist-hip circumference ratio	0.85 ± 0.11	0.88 ± 0.07	<0.001	16.12 (6.28, 41.35)	0.037	0.31 (0.10, 0.93)
Heart rate	74.8 ± 10.0	74.2 ± 10.0	0.098	0.99 (0.99, 1.001)	0.363	0.99 (0.99, 1.00)
Systolic blood pressure (mmHg)	126.8 ± 17.9	137.4 ± 19.8	<0.001	1.03 (1.025, 1.03)	0.088	0.99 (0.99, 1.00)
Diastolic blood pressure (mmHg)	76.6 ± 11.1	79.0 ± 10.8	<0.001	1.02 (1.01, 1.02)	0.564	1.002 (0.99, 1.01)
Sodium (mmol/L)	139.6 ± 2.1	140.4 ± 1.9	<0.001	1.24 (1.19, 1.29)	<0.001	1.12 (1.07, 1.16)
Potassium (mmol/L)	4.18 ± 0.30	4.21 ± 0.31	0.001	1.50 (1.19, 1.91)	0.243	0.87 (0.68, 1.10)
Calcium (mmol/L)	2.34 ± 0.09	2.33 ± 0.09	0.009	0.34 (0.15, 0.76)	0.951	1.03 (0.44, 2.38)
Phosphate (mmol/L)	1.13 ± 0.15	1.12 ± 0.15	0.030	0.59 (0.36, 0.95)	0.174	1.42 (0.86, 2.34)
Blood urea nitrogen (mmol/L)	4.73 ± 1.27	5.16 ± 1.29	<0.001	1.23 (1.18, 1.29)	0.011	0.93 (0.88, 0.98)
Uric acid (μmol/L)	328.6 ± 87.0	324.4 ± 81.0	0.164	0.99 (0.99, 1.00)	0.004	0.99 (0.99, 0.99)
Estimated glomerular filtration rate (ml/min/1.73 m^2^)	102.8 ± 14.8	88.4 ± 13.0	<0.001	0.95 (0.94, 0.95)	0.047	1.005 (1.001, 1.01)
Blood glucose (mmol/L)	5.34 ± 1.26	5.70 ± 1.29	<0.001	1.15 (1.11, 1.19)	0.042	0.94 (0.89, 0.99)
High sensitive C-reactive peptide (mg/L)	1.44 ± 3.14	1.77 ± 4.83	0.006	1.02 (1.006, 1.04)	0.662	0.99 (0.97, 1.02)
Lipoprotein a (mg/L)	19.6 ± 22.1	23.5 ± 24.4	<0.001	1.007 (1.004, 1.01)	0.057	1.00 (1.00, 1.01)
Triglyceride (mmol/L)	1.36 ± 1.20	1.50 ± 1.12	0.001	1.07 (1.03, 1.12)	0.479	1.02 (0.96, 1.09)
Total cholesterol (mmol/L)	4.89 ± 0.94	5.15 ± 0.97	<0.001	1.29 (1.21, 1.38)	<0.001	1.14 (1.07, 1.23)
Low density lipoprotein (mmol/L)	2.89 ± 0.78	3.08 ± 0.83	0.000	1.31 (1.21, 1.42)	0.001	1.16 (1.07, 1.26)
High density lipoprotein (mmol/L)	1.45 ± 0.40	1.48 ± 0.41	0.112	1.15 (0.97, 1.36)	0.070	1.18 (0.99, 1.40)
Arterial hypertension (*n*/%)	1,117/5.2	121/14.5	<0.001	3.11 (2.54, 3.81)	0.189	0.86 (0.69, 1.08)
Diabetes mellitus (*n*/%)	1,858/8.5	142/16.9	<0.001	2.19 (1.82, 2.64)	0.033	0.81 (0.66, 0.98)

*Data are presented as mean ± standard deviation for continuous variables, and as the number of cases/percentages for categorical variables, respectively*.

*OR, odds ratio; CI, confidence interval*.

*>All variables in the univariable analysis were further adjusted by age*.

*Two-tailed values of P < 0.05 were considered statistically significant*.

**Table 3:** Risk factors associated with primary epiretinal membranes by multivariable analysis.

	**All**		**Men**		**Women**	
	***P-*value**	**OR (95% CI)**	***P*-value**	**OR (95% CI)**	***P*-value**	**OR (95% CI)**
Age (year)	<0.001	1.10 (1.10, 1.11)	<0.001	1.11 (1.10, 1.13)	<0.001	1.11 (1.09, 1.13)
Women (%)	0.341	1.10 (0.91, 1.33)	/	/	/	/
Waist-hip circumference ratio	0.737	0.86 (0.34, 2.14)	0.559	0.51 (0.05, 5.01)	0.638	1.67 (0.20, 14.16)
Systolic blood pressure (mmHg)	0.498	0.998 (0.993, 1.003)	0.530	1.002 (0.99, 1.01)	0.221	0.99 (0.99, 1.003)
Sodium (mmol/L)	<0.001	1.12 (1.07, 1.17)	0.369	1.04 (0.96, 1.12)	<0.001	1.18 (1.10, 1.27)
Blood urea nitrogen (mmol/L)	0.076	0.94 (0.88, 1.01)	0.684	0.98 (0.88, 1.08)	0.596	0.97 (0.88, 1.08)
Urine acid (μmol/L)	0.644	1.00 (0.998, 1.001)	0.227	1.001 (0.99, 1.003)	0.156	0.99 (0.99, 1.001)
Blood glucose (mmol/L)	0.256	0.96 (0.89, 1.03)	0.119	0.90 (0.79, 1.03)	0.644	1.03 (0.90, 1.19)
Lipoprotein a (mg/L)	0.880	1.00 (0.997, 1.004)	0.505	1.002 (0.99, 1.01)	0.788	0.99 (0.99, 1.004)
Total cholesterol (mmol/L)	0.003	1.14 (1.05, 1.24)	0.328	1.07 (0.93, 1.24)	0.031	1.15 (1.01, 1.30)
High density lipoprotein (mmol/L)	0.122	1.19 (0.96, 1.48)	0.590	0.89 (0.59, 1.36)	0.293	1.19 (0.86, 1.65)
Estimated glomerular filtration rate (ml/min/1.73m^2^)	0.050	1.01 (1.00, 1.02)	0.106	1.01 (0.99, 1.03)	0.721	1.002 (0.99, 1.02)
Diabetes mellitus (%)	0.977	1.01 (0.73, 1.40)	0.157	1.38 (0.88, 2.14)	0.159	0.70 (0.43, 1.15)

*OR, odds ratio; CI, confidence interval; Low-density lipoprotein was not included as it shares the co-linearity with the total cholesterol (variance inflation factors > 5)*.

*Two-tailed values of P < 0.05 were considered statistically significant*.

**Figure 1:**
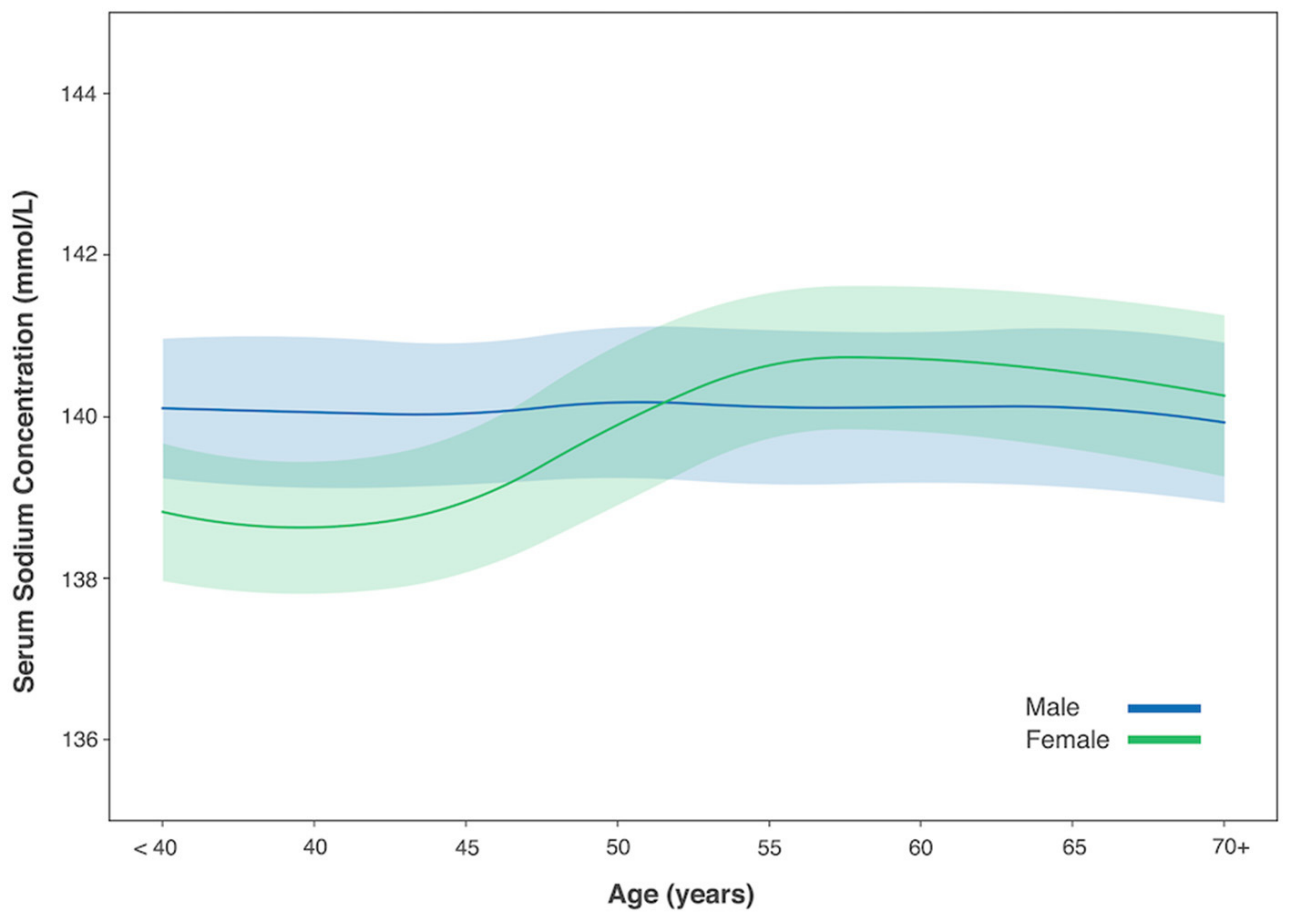
Serum sodium concentration with age in male and female gender. Figure was shown with mean value (solid line) and 1/2 standard deviation (shadow).

The mean SSC was 139.6 ± 2.1 mmol/L (range: 120–150 mmol/L) measured in 21,054 participants, with 30 (0.1%) individuals with hypernatremia and 250 (1.2%) participants with hyponatremia. In individuals with normal SSC (135–145 mmol/L), a positive correlation between higher SSC and older age was found in women (*r* = 0.33, *P* < 0.001, Pearson correlation analysis) but not in men (*P* = 0.35). In women, SSC increased continuously from the age of 50 years to the age of 70 years (one-way ANOVA, *P* < 0.05), after which it dropped slightly. The SSC did not differ significantly among the age groups in men (one-way ANOVA, *P* = 0.18) (Table 4; Figure 1).

**Table 4:** Serum sodium concentration in the study population, stratified by age and sex (with serum sodium concentration within the normal range).

**Age group (years)**	**All**		**Men**		**Women**		***P*-value (men vs. women)**
	***n***	**Sodium (mmol/L)**	***n***	**Sodium (mmol/L)**	***n***	**Sodium (mmol/L)**	
<40	9,949	139.4 ± 1.8	4,055	140.1 ± 1.7	5,394	138.8 ± 1.7	<0.001
40–49	4,268	139.4 ± 1.9	1,943	140.1 ± 1.8	2,325	138.8 ± 1.7	<0.001
50–59	3,666	140.2 ± 1.9^*^^†^	1,671	140.1 ± 1.9	1,995	140.3 ± 1.9^*^^†^	0.032
60–69	2,479	140.4 ± 1.9^*^^†^^‡^	1,133	140.1 ± 1.9	1,346	140.6 ± 1.8^*^^†^^‡^	<0.001
70+	912	140.1 ± 2.0^*^^†^^#^	423	139.9 ± 2.0	489	140.2 ± 2.0^*^^†^^#^	0.089
Total	20,774	139.7 ± 1.9	9,225	140.1 ± 1.8	11,549	139.3 ± 1.9	<0.001

*The concentrations of serum sodium are displayed as mean ± standard deviation*.

*Serum sodium concentrations between the men and women were compared by independent t-test*.

*Serum sodium concentrations among different age groups were compared by one-way analysis of variance, and post hoc tests adjusted by Bonferroni were performed when a significant difference was detected*.

*Two-tailed values of P < 0.05 were considered statistically significant*.

* *Compared with participants <40 years old, P < 0.05*.

† *Compared with participant age 40–49, P < 0.05*.

‡ *Compare with participants aged 50–59, P < 0.05*.

# *Compared with participants aged 60–69, P < 0.05*.

*Note there was a decrease in sodium level in people aging 70+ years*.

**Figure 2:**
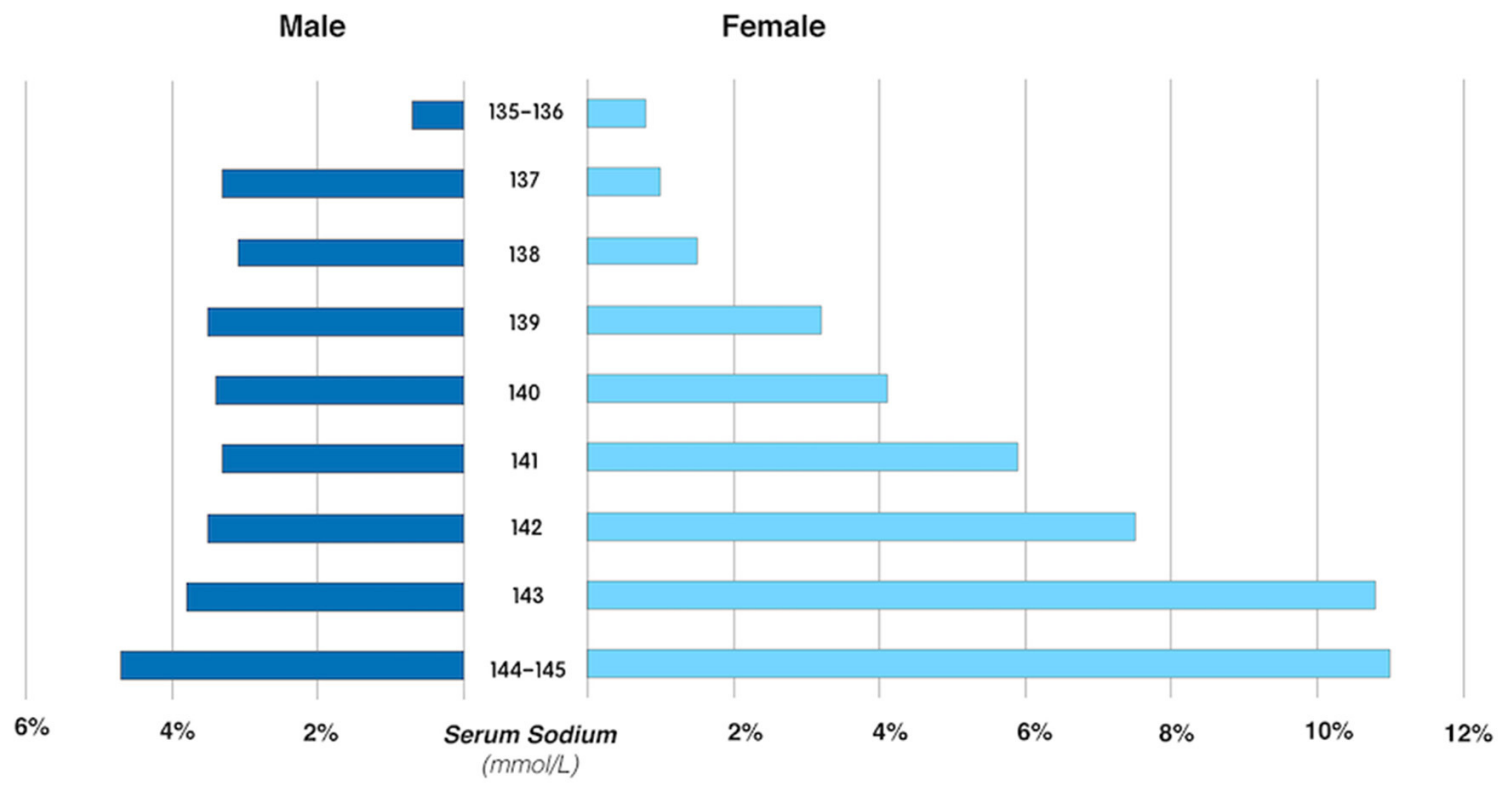
The prevalence of primary epiretinal membrane in male and female participants, stratified by the serum sodium concentration.

The association between SSC and ERM was modified by sex in the univariable analysis (*P* for interaction <0.001). In multivariable analysis, the association between SSC and ERMs remained statistically significant in the whole study population and in women (both *P* < 0.001), but not in men (both *P* > 0.05) (Tables 3, 5). In women, the ERM prevalence significantly increased from 0.9% in the subgroup with SSC of 135–137 mmol/L, to 3.0% (SSC: 138–140 mmol/L), 7.1% (SSC: 141–143 mmol/L) and to 11.0% (SSC: 144–145 mmol/L) (linear-by-linear association chi-square test, *X*^2^ = 160.04, *P* < 0.001). Female participants with an SSC of 144–145 mmol/L as compared with women with an SSC of 135–137 mmol/L had a 5.3-fold increased risk of ERMs (OR: 5.33; *P* < 0.001) in the multivariable analysis (Figure 2; Table 6).

**Table 5:** Serum sodium concentration in study participants with vs. without primary epiretinal membranes (ERMs) (with serum sodium concentration within the normal range).

	**All**	**Primary ERMs**	**No ERMs**	**Crude analysis**		**Age-adjusted analysis**		Multivariable analysis^*^	
				***P*-value**	**OR (95%CI)**	***P*-value**	**OR (95%CI)**	***P*-value**	**OR (95%CI)**
Men	140.1 ± 1.8 (9,105)	140.3 ± 1.8 (306)	140.1 ± 1.8 (8,799)	0.07	1.06 (0.99, 1.13)	0.026	1.08 (1.01,1.15)	0.364	1.04 (0.96, 1.13)
Women	139.3 ± 1.9 (11,422)	140.6 ± 1.8 (436)	139.3 ± 1.9 (10,986)	<0.001	1.41 (1.34,1.49)	<0.001	1.15 (1.09, 1.21)	<0.001	1.19 (1.11, 1.28)
All	139.7 ± 1.9 (20,527)	140.5 ±1.8 (742)	139.6 ± 1.9 (19,785)	<0.001	1.25 (1.21,1.30)	<0.001	1.12 (1.07, 1.16)	<0.001	1.12 (1.07, 1.18)

* *Adjusted by age, waist-hip circumference ratio, blood urea nitrogen, urine acid, estimated glomerular filtration rate, glucose, lipoprotein a, total cholesterol and high-density lipoprotein as continuous variables, and the presence of diabetes mellitus and sex (except in sex-specific analysis) as binary variables*.

*OR, odds ratio; CI, confidence interval*.

*The concentrations of serum sodium are presented as mean ± standard deviation (number of individuals)*.

*Two-tailed values of P < 0.05 were considered statistically significant*.

**Table 6:** Association between the prevalence of primary epiretinal membranes (ERMs) and the serum sodium concentration by multivariable analysis, stratified by sex.

**Sodium Level (mmol/L)**	**Total (*****n*** **=** **20,661)**			**Women (*****n*** **=** **11,494)**			**Men (*****n*** **=** **9,167)**		
	***N***	**ERMs (%)**	OR (95% CI)*	***n***	**ERM (%)**	OR (95% CI)*	***n***	**ERM (%)**	OR (95% CI)*
135–137	2,678	1.3 ± 0.2	Reference	1,957	0.9 ± 0.2	Reference	721	2.4 ± 0.6	Reference
138–140	11,101	3.1 ± 0.2	2.29 (1.52, 3.46)	6,495	3.0 ± 0.2	2.76 (1.51, 5.01)	4,606	3.3 ± 0.3	1.69 (0.92, 3.09)
141–143	6,439	5.0 ± 0.3	2.478 (1.64, 3.75)	2,833	7.1 ± 0.5	3.45 (1.85, 6.44)	3,606	3.4 ± 0.3	1.52 (0.80, 2.89)
144–145	443	7.7 ± 1.5	3.44 (1.99, 5.95)	209	11.0 ± 2.2	5.33 (2.41, 11.79)	234	4.7 ± 1.7	1.75 (0.70, 4.39)
*P*-value			<0.001			<0.001			0.363

* *Adjusted by age, waist-hip circumference ratio, blood urea nitrogen, urine acid, estimated glomerular filtration rate, glucose, lipoprotein a, total cholesterol and high-density lipoprotein as continuous variables, the presence of diabetes mellitus and sex (except in sex-specific analysis) as binary variables*.

*OR, odds ratio; CI, confidence interval*.

*The prevalence rate of primary ERMs displayed as mean ± standard error*.

*Two-tailed values of P < 0.05 were considered statistically significant*.

*A significantly linear association between higher SCC and the ERMs prevalence was detected in women (linear-by-linear association chi-square test, X^2^ = 160.04, P < 0.001), but not in men (X^2^ = 2.238, P = 0.137)*.

**Figure 3:**
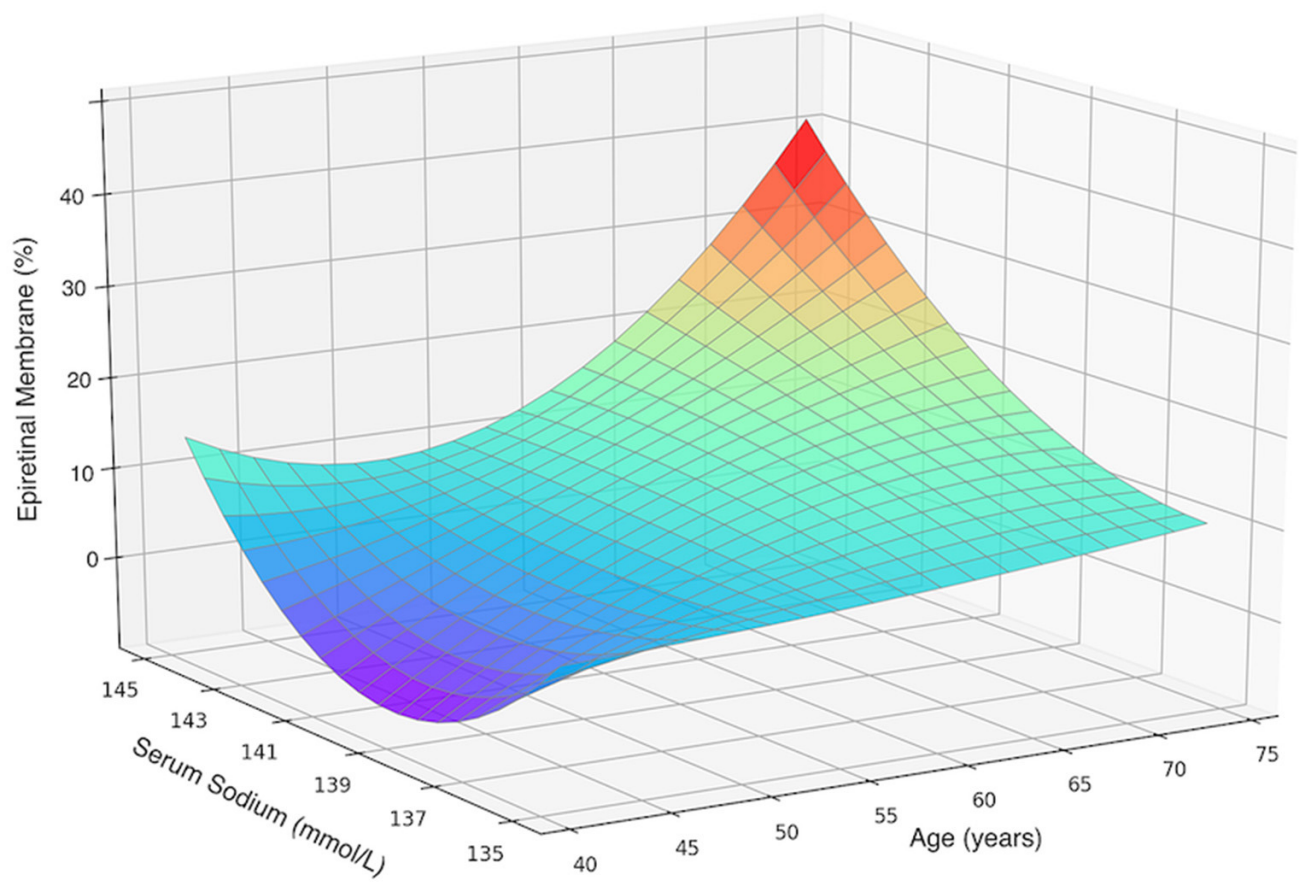
Three-dimensional surface plot visualizing the functional associations between the presence of primary epiretinal membrane and two dependent variables including age and serum sodium concentration in female participants aged 40 and over. An increase in epiretinal membrane prevalence with an increased sodium level was not dependent on age.

If only participants aged 40+ years were included, the SSC was again significantly (*P* < 0.001) higher in women with primary ERMs (140.6 ± 1.8 mmol/L) as compared with women without ERMs (139.7 ± 2.0 mmol/L). Women with SSC of 144 mmol/l to 145 mmol/L as compared to women with an SSC of 135–137 mmol/L had 4.6-folds increased risk of ERMs (OR: 4.63; 95% CI: 2.10–10.21; *P* < 0.001) (Figure 3).

In a next step, all female patients with ERM were compared with 1:1 age-matched female controls, for a sensitivity analysis. A significant higher SSC was found in ERM patients (*n* = 436, age: 62.0 ± 8.4 years, SSC: 140.6 ± 1.8 mmol/L) in comparing with age-matched controls (*n* = 436, age: 62.0 ± 8.5 years, SSC: 137.5 ± 1.3 mmol/L) after multivariable analysis (OR: 6.39; 95% CI: 4.61–8.88; *P* < 0.001).

When comparing the subtypes of ERMs, female participants with PMFs as compared to those with CMR also show a slightly but significantly higher SSC (140.8 ± 1.8 vs. 140.3 ± 2.0 mmol/L; *P* = 0.01).

Inter-observer agreement was assessed by examining the photographs of 100 participants by both examiners (CCX, XBZ), with a kappa value of 0.851 (*P* < 0.001).

## Discussion

The prevalence of primary ERMs in our study population was 3.7% and increased with older age. Factors associated with a higher ERM prevalence were, besides older age, a higher serum concentration of total cholesterol and a higher SSC. If the study population was stratified by sex, the correlation between a higher SSC, even within the normal range, with a higher ERM prevalence, was valid for women but not for men. Women with an SSC of 144–145 mmol/L as compared with those with an SSC of 135–137 mmol/L had a 5.3-fold increased odds of having an ERM for the whole age group, and 4.6-fold increased odds of having an ERM for those aged 40+ years.

The prevalence of primary ERMs as found in our study population was higher than the ERM frequency observed in other Asian populations (4, 5, 7, 16), but was very comparable to the pooled data of a recent meta-analysis (6).

Increasing age is a well-established risk factor for ERM development, as consistently reported by previous population-based studies and ours (1, 5, 17–19). One may discuss that vitreoretinal interface changes caused by a posterior vitreous detachment may be causally associated with the age-related increase of the ERM prevalence.

The association between female gender and higher ERM prevalence was inconsistently reported. While in a recent meta-analysis pooled 13 studies with 49,696 participants, female individuals tended to have a higher ERM prevalence than men with an OR of 1.34 (6), many investigations as well as ours did not find a sex-specific difference in the ERM prevalence (5, 7, 20, 21). The discrepancies in the association between sex and ERM may be due to variations of the ethnicities, study designs, and techniques to detect ERM.

The new finding in our study was the relationship between a higher SSC and a higher primary ERM prevalence, including or excluding participants with an abnormal SSC. In a case series study, sodium was detected in 10 out 20 ERM samples and tended to be more frequent in the thicker ERM (22), which is consistent with the significantly higher SSC in PMF patients than CMR patients in our study. In a study involving 27 participants with a macular hole or ERM, the sodium concentration in the vitreous was significantly higher than that in the fasting serum samples obtained perioperatively (146.7 ± 3.3 vs. 139.7 ± 3.4 mmol/L; *P* < 0.0001) (23).

Our findings combined with these studies suggest that sodium may be a factor involved in the pathogenesis of ERM, perhaps by modulating the glial activity and cell migration (22–25), whereas the exact mechanism remains to be explored. A cross-sectional study from Korea observed no difference in sodium intake between participants with and without ERM (16). One may take into account, however, that their data of the sodium intake were self-reported and not equivalent to the SSC.

Sodium (Na+) is a major electrolyte in the serum and extracellular fluids. Both higher and lower SSC, even within the normal range (135–145 mmol/L), are associated with primary cardiovascular events (26). A higher SSC is related to elevated serum lipid concentrations and elevated blood pressure, and extracellular sodium directly affects lipid metabolism by increasing the lipid accumulation in cultured adipocytes (27). Thus, the association between higher cholesterol and ERM prevalence observed in our study and previous similar findings (4, 8, 10, 18) may also help to explain the relationship between a higher SSC and higher ERM prevalence.

A positive correlation between SSC and age was found in women but not in men. The SSC in women was lower than that in men up to the age of 50 years, while beyond 50 years the association even reversed. This tendency has also been previously observed (28, 29). Besides, the female gender was also found to be an independent risk factor for hyponatremia (30–32). Possible explanations for these sex-specific differences may be the impact of female sex hormones in regulating serum sodium, sex differences in regulation of arginine vasopressin, renal sodium secretion, and absorption (28, 29, 33, 34). Interestingly, menopause among Chinese women occurs at approximately the age of 50 years, consisting with the age when the difference in SSC between women and men started to reverse.

Notably, though the age-related variations in SSC might have functioned as confounding factors, the significant association between SSC and ERM prevalence remained after age and multivariable adjustment, in all female participants and in those age 40+ years. It was also shown in a three-dimensional surface plot in which the ERM prevalence increases with the SSC, after stratification by age (Figure 3). Our findings may help to explain the higher ERM prevalence in women than men detected by other groups.

The strengths of our study include its large sample size, and the relatively large number of systemic parameters included in the statistical analysis. These parameters included the blood pressure, glucose, lipids and kidney function, and presence of hypertension and diabetes, which may be potential confounding factors for any association with SSC. Our study has several limitations. First, a selection bias may exist since our results were not derived from a population-based sample. However, most of our participants were employees or retirees from fixed organizations and were consecutively enrolled without considering their health conditions or other factors. Second, the lack of optical coherence tomography might have led to an underestimation of the ERM prevalence. However, fundus photography, as also adopted by other groups (1, 3, 4), has the advantage of a wider angle of imaging so that the identification of ERMs at the temporal vascular arcades became easier. In addition, the primary ERM prevalence in our study was comparable to that reported in a recent meta-analysis (6). Third, the medication and dietary information were not available for the current study, thus we are not sure about the causes of high SSC in individuals with ERM. Whether a low-salt diet would prevent the development or progression of ERM might be of interest to explore in the future. Fourth, our study is a cross-sectional investigation that could assess only factors associated with the prevalence of ERM but not risk factors for the incidence of ERM.

In conclusion, besides older age and higher total cholesterol, a higher serum sodium level may be an independent factor associated with the presence of ERM.

## Data Availability Statement

The raw data supporting the conclusions of this article will be made available on reasonable request from the corresponding authors.

## Ethics Statement

The Institutional Review Board of Beijing Tongren Hospital approved the study design and waived the informed consent, under the measures for ethical reviews of biomedical research involving humans initiated by the Chinese government and consistent with the FDA guidance.

## Author Contributions

YW and JJ: design of the study and critical review of manuscript. CX, JC, XZ, and JX: conduct of the study and collection and management of the data. CX, JC, CZ, DC, and YW: analysis and interpretation of data. CX, JC, and YW: preparation of manuscript. All authors provided important feedback on the methods and results and approved the final version of the manuscript.

## Funding

The work was funded by the Research Development Fund of Beijing Municipal Health Commission (2019–4). The funder of this study had no role in study design, data collection, data analysis, data interpretation, or writing of the manuscript.

## Conflict of Interest

The authors declare that the research was conducted in the absence of any commercial or financial relationships that could be construed as a potential conflict of interest.

## Publisher's Note

All claims expressed in this article are solely those of the authors and do not necessarily represent those of their affiliated organizations, or those of the publisher, the editors and the reviewers. Any product that may be evaluated in this article, or claim that may be made by its manufacturer, is not guaranteed or endorsed by the publisher.

## Abbreviations

ERM: epiretinal membrane
SSC: serum sodium concentration
CMR: cellophane macular reflex
PMF: pre-macular fibrosis.
